# How many people have been bitten by dogs? A cross-sectional survey of prevalence, incidence and factors associated with dog bites in a UK community

**DOI:** 10.1136/jech-2017-209330

**Published:** 2018-02-01

**Authors:** Carri Westgarth, Megan Brooke, Robert M Christley

**Affiliations:** 1 Department of Epidemiology and Population Health, Institute of Infection and Global Health, University of Liverpool, Cheshire, UK; 2 Institute of Veterinary Science, University of Liverpool, Cheshire, UK

**Keywords:** dogs, canine, bites, epidemiology, community surveys

## Abstract

**Background:**

Dog bite studies are typically based on hospital records and may be biased towards bites requiring significant medical treatment. This study investigated true dog bite prevalence and incidence at a community-level and victim-related risk factors, in order to inform policy and prevention.

**Methods:**

A cross-sectional study of a community of 1280 households in Cheshire, UK, surveyed 694 respondents in 385 households. Data included dog ownership and bite history, demographics, health and personality (Ten Item Personality Inventory (TIPI) brief measure). Multivariable logistic regression modelled risk factors for having ever been bitten by a dog, accounting for clustering of individuals within households.

**Results:**

A quarter of participants (24.78%, 95% CI 21.72 to 28.13) reported having ever been bitten by a dog during their lifetime, with only a third of bites described requiring further medical treatment and 0.6% hospital admission. Incidence of dog bites was 18.7 (11.0–31.8) per 1000 population per year. Males were 1.81 times more likely to have been bitten in their lifetime than females (95% CI 1.20 to 2.72, P=0.005). Current owners of multiple dogs were 3.3 times more likely (95% CI 1.13 to 9.69, P=0.03) to report having been bitten than people not currently owning a dog. Regarding all bites described, most commonly people were bitten by a dog that they had never met before the incident (54.7%). Individuals scoring higher in emotional stability had a lower risk of having ever been bitten (OR=0.77 for 1 point change in scale between 1 and 7, 95% CI 0.66 to 0.9, P=0.001).

**Conclusion:**

This study suggests that the real burden of dog bites is considerably larger than those estimated from hospital records. Further, many bites do not require medical treatment and hospital-based bite data are not representative of bites within the wider population. Victim personality requires further investigation and potential consideration in the design of bite prevention schemes.

## Introduction

With a dog population of around 8.5 million^1^ in the UK (ie, 1 for every 7–8 people in a population of 65 million)^2^ dog bites are bound to occur.^3^ However, the rate of dog bite occurrence remains unknown,^4^ which has implications for knowledge of the true burden on public health and economic loss to the health system. There are an estimated 6743 hospital admissions for ‘dog bites and strikes’ per year in England^5^ and 9500 for dog bites in USA.^6^ Dog bite rates are also thought to be rising.^6 7^ However, these figures actually concern ‘bites and strikes’ from a dog (strikes meaning injury caused by a dog but not from a bite), yet are commonly misreported as dog bites.^4^ Statistics concerning actual dog bites require clarification. Further, prevalence studies based on hospital admission records do not include likely less serious dog bites which do not require medical treatment or are treated elsewhere, including Accident and Emergency.^4 8 9^ In a population-based study in USA over 20 years ago, six times more bites occurred than required medical attention.^10^ The current often cited incidence for UK is estimated to be around 2.5 per 100 000 requiring hospital admission and 740 per 100 000 bitten.^11^ However, these figures are based on out-of-date data, incorrectly cited or even no true source is known^4^ and medical literature has a tendency to exaggerate dog bite risk.^12^ Given the implications of even minor dog bites to both physical health^13^ (including rabies in some countries)^14^ and psychological health,^15 16^ newer and more accurate figures are essential.

With 21 fatal UK dog attacks occurring within 10 years, pressure has been put on governments to enact more control measures and protect the public.^17^ Experts feel that there is a lack of understanding as to how victim/owner behaviour and misunderstanding of dog signalling can provoke dog bites, and they also have significant doubt as to the effectiveness of current dog bite reduction legislation.^3 18–20^ The UK Dangerous Dogs Act (1991) has recently been extended such that dog owners can be also prosecuted for bites occurring on private property.^21^ This was in our view sensible and due to expert opinion and evidence that most bites occur from a familiar dog in familiar surroundings;^18 22 23^ however, other studies do contradict this view.^24 25^ New data are required in order to evidence whether people are generally bitten by familiar or unfamiliar dogs, as bite prevention strategies may need to be approached quite differently for different contexts and the importance of both contexts addressed adequately in prevention initiatives.

The first aim of this study was to provide a community population-level estimate for dog bite prevalence (how many people report ever having been bitten by a dog) and incidence (rate of new dog bites per year). The second aim was to estimate the proportion of dog bites that require medical treatment and if so, where treatment was sought. The third aim of this study was to determine victim-related factors associated with having ever been bitten by a dog at the population level. Hypothesised variables of importance based on previous research in other population types (such as hospital based data) and geographical locations included age and gender.^26^ Others hypothesised to be important included: current dog ownership, as this is likely to increase opportunity for interactions with dogs; education level, as socioeconomic factors can influence public health outcomes such as risk of injuries; general health, as health conditions may influence the nature of interactions with dogs; reasons for getting a dog, as this may again influence the type of interactions that occur with the dog and personality, due to expert opinion that victim behaviour around dogs is important and behaviour may be influenced by personality. The fourth aim was to identify the most common relationships between victims and the dogs that bit them.

## Methods

A cross-sectional census study of 1280 households was conducted between June and August 2015 in part of a semirural town in Cheshire West. This area was chosen because of its mixture of housing types, definition by natural and man-made boundaries and convenience as a research location and has been used for previous research.^27 28^ Attempts were made to survey all households within the study area by knocking on their door up to five times. The interviewers were female veterinary students, personable and briefed in friendly interview technique. Contact was made with 984 households (76.9%) and 217 (22.1%) declined to participate. For 767 households (77.9%), a brief survey was completed at the door asking about current and past dog ownership. Further paper questionnaires were provided for all household members; a different questionnaire was issued for adults and children (5–15 years). There was also an option to complete the questionnaire online. In total, 698 households (91.0% of those door-step interviewed) were left with questionnaires for 1591 eligible participants aged over 5 years. Questions related to the individuals’ health, exercise, dog ownership and dog bites as well as collecting demographic information. Postcard reminders were sent at 2 weeks and a second copy of the questionnaire at 4 weeks. The data for this paper were collected as part of a wider study comparing health and physical activity of dog owners and non-dog owners, for which children less than 5 years were not included due to difficulties in studying these outcomes using even parental surveys. The study conformed to the principles embodied in the Declaration of Helsinki.

### Outcomes

Participants were asked to report how many times they had been bitten by a dog (if at all) and whether a dog bite occurred in the last year (yes/no). Participants were asked to choose one bite event to provide further information on. This included: whether the participant knew the dog (No, I did not know the dog previous to this occasion/Yes, but only briefly (seen on walks and so on)/Yes it was a well know friend/family members dog/Yes, it was my own dog); how old they were at the time of the bite (years) and whether they required medical treatment from a doctor or hospital (yes/no) and where from (Hospital-admitted/Accident and Emergency/Walk in Centre/GP Surgery/Other).

### Variables

Demographic data collected included: current age (calculated from year of birth); gender (male/female) and highest education level (16 options). Education was later categorised into four categories (see table 1). Participants were asked how many dogs they currently owned. Participants who owned dogs were also asked to indicate their (multiple) reasons were for getting a dog: (companionship/protection/ interest/hobby/gift/ to show/to breed/exercise/working dog/always had a dog/family member wanted a dog/other). Dog owners were also asked whether they usually participated in walking the dog (yes/no). All respondents were asked to rate their general health on a five-point scale of poor to excellent. ‘Big Five’ personality traits were assessed for all adults using the validated 10-Item Personality Inventory,^29^ on a scale of 1–7.

**Table 1:** Demographic information for 694 individuals within the study sample

Variable	Number	Percentage	Mean	Median
Gender				
Male	319	46.0		
Female	375	54.0		
Missing	0			
Ownership status				
Non-dog owner	494	71.2		
Dog owner	200	28.8		
Missing	0			
Number of dogs owned				
1	175	87.5		
2	20	10. 0		
3	3	1.5		
6	2	1.0		
Missing	0			
Age				
5–15	48	6.9		
16–30	63	9.1		
31–45	87	12.5		
46–60	176	25.4		
61–75	244	35.2		
76+	62	8.9		
Missing	14			
Highest education				
Degree/diploma or higher professional qualification	303	48.6		
A level equivalent	70	11.2		
GCSE or O’level equivalent	149	23.9		
Other school certificate or none	101	16.2		
Missing	71			
Perceived general health				
Poor	31	4.5		
Fair	95	13.7		
Good	243	35.0		
Very Good	220	31.7		
Excellent	92	13.3		
Missing	13			
Personality (adult)				
Extroverted	588 completed		4.26	4
Conscientiousness	591 completed		5.74	6
Open to new experiences	589 completed		4.9	5
Agreeableness	589 completed		5.39	5.5
Emotionally stability	592 completed		5.06	5

### Analysis

Confidence intervals for sample proportions were estimated using the Wilson method in EpiTools.^30^ Missing data were excluded from analysis. The association between categorical variables, such as demographic factors and bite-related outcomes was assessed using χ²  analysis. Univariable binary logistic regression analysis was also conducted to assess associations between variables and the primary outcome of whether the person had ever been bitten by a dog. Variables which had a P value of 0.250 or lower were used to construct a multivariable model and a backwards stepwise selection process was conducted until all remaining variables were significant at P<0.05. Multivariable mixed effects logistic regression analysis was used to explore the association between explanatory variables and the outcome variable, while accounting for clustering of participants within households. Models were built for outcome ever been bitten by a dog (Model A) and on a subset excluding those who reported details of a bite that occurred over 5 years ago (Model B). Analysis was conducted using the R Language for Statistical Computing,^31^ including the lme4 package for mixed effects modelling.

## Results

### Sample characteristics

A total of 694 (43.6%) participants returned their questionnaires, from 385 (55.2%) households that were given questionnaires to return, out of a total sample of 1280 households (total household response rate 30.1%). The sample characteristics for the population completing the survey are outlined in table 1. There were slightly more female than male participants and the majority of individuals within the study were of an older age.

### Description of dog bites reported

A quarter (24.78%, 95% CI 21.72 to 28.13, n=172) of the respondents had been bitten by a dog on at least one occasion. In total, 301 bites were reported. Only 13 (1.87%, 95% CI 1.10 to 3.18) individuals had been bitten in the last 12 months. This equates to a dog bite incidence of 18.7/1000 per year (95% CI 11.0 to 31.8). Of the individuals who reported ever being bitten (172), 57.6% had only been bitten once (mean 1.75, range 9). Only 33.1% of the dog bites described required any medical treatment. The number of times individuals had been bitten did not alter this figure, and there was no evidence of an association between medical treatment and gender (P=0.412). To summarise where medical treatment occurred if it was sought: 33 (58.9%) A&E; 17 (30.3%) general practitioner; 4 (7.1%) walk-in centre; 1 (1.8%) hospital admission and 1 (1.8%) police station.

Forty-four per cent of the bites described occurred in childhood (<16 years). However, of the 48 children surveyed (aged 5–15) within the study only three had been bitten (6.3%). All other reports of childhood bites were retrospective as adults and many (69.2%) described a bite that occurred more than 15 years ago. Over half (54.7%) of people were bitten by dogs they had never met before. Of the 87 individuals who had been bitten only once, 51 (58.6%) reported not knowing the dog, indicating that this finding is not due to reporting bias. There was no evidence that the relationship with the dog that bit varied by current dog ownership, whether medical treatment was sought, victim age or gender (table 2).

**Table 2:** The relationship between bite victims and the dog that bit them, split by victim gender (P=0.228)

Did you know the dog that bit you?	Victim gender	
	Male	Female
No, I did not know the dog previous to this occasion	56 (59.6%)	39 (48.7%)
Yes, but only briefly (seen on walks and so on)	9 (9.6%)	7 (9.2%)
Yes, it was a well-known friend’s/family members’ dog	13 (13.8%)	20 (26.3%)
Yes, it was my own dog	16 (17%)	12 (15.8%)
Total	94 (100%)	76 (100%)

### Factors associated with having ever being bitten by a dog

Univariable analysis findings and numbers reported by bitten/never bitten are presented in the online supplementary file. Current age risk was found to have a non-linear shape, with increasing risk that plateaued at approximately age 50. Therefore for final model building of Model A, the upper age was limited to 50 (all ages above this recoded to 50), so that the risk pertaining to cumulative age could be included and modelled in a linear manner.

10.1136/jech-2017-209330.supp1 Supplementary file

On multivariable analysis (table 3), for model A (ever bitten by a dog), males were 1.81 times more likely to have ever been bitten than females (95% CI 1.20 to 2.72, P=0.005). Multiple dog owners were 3.31 times more likely to have ever been bitten than non-dog owners (95% CI 1.13 to 9.69, P=0.03). Odds of ever being bitten increased with age (up to 50) by 1.03 (95% CI 1.01 to 1.06) per year (P=0.01), which is unsurprising as this measures cumulative risk over lifespan to date and an important adjustment to include in the model. Scoring as more emotionally stable/lower neuroticism by one a point change in score (between 1 and 7) decreased the likelihood of experiencing a bite by 0.77 times (95% CI 0.66 to 0.90, P=0.001). Similar variables were found to be associated in Model B where those reporting being bitten in the past 5 years (28 participants) were analysed against those reporting never having been bitten by a dog. However, the findings of this model should be interpreted with caution due to small sample size and the fact that it will have excluded people who were bitten more than once but chose to report details of a different bite than the one that occurred during the past 5 years, as we could not tell if they were bitten in the past 5 years also. However the findings reassure that gender, number of dogs and personality-emotional stability are important factors. Current age was this time not significant, not surprising given that cumulative lifetime risk is not important given we are only modelling bites in the last 5 years. However, it also does not provide evidence that current age (near to the time of the bite) was associated with bite risk.

**Table 3:** Final multivariable models of factors associated with having ever been bitten by a dog (Model A) and reporting a bite that occurred in the last 5 years (Model B), including random effect of clustering of participants within households (n=578 and 456, respectively)

Variable	Model A		Model B	
	OR (95% CI)	P value	OR (95% CI)	P value
Gender				
Female	1.00		1.00	
Male	1.81 (1.20 to 2.72)	0.005	5.05 (5.01 to 5.09)	<0.001
Current age				
Per year (Model A age limited at 50)	1.03 (1.01 to 1.06)	0.01	1.0 (0.99 to 1.00)	0.40
Number of dogs				
Owns no dogs	1.00		1.00	
Owns one dog	1.58 (0.99 to 2.52)	0.54	9.58 (9.50 to 9.65)	<0.001
Owns multiple dogs	3.31 (1.13 to 9.69)	0.03	27.71 (2.75 to 2.79)	<0.001
Personality (TIPI)				
Emotional stability* scale	0.77 (0.66 to 0.90)	0.001	0.66 (0.65 to 0.66)	<0.001

*Emotional stability also known as neuroticism.

## Discussion

This study provides much improved indicators of the true burden of dog bites on public health. One in four people had ever been bitten by a dog and when a bite occurred, only one-third sought any form of medical treatment. Only 1 of the 172 bites (0.6%) reported in more detail resulted in hospital admission; given the small sample size and self-selection of which bites to describe further, this finding must be interpreted with caution but may suggest that figures based on hospital admission records (eg, 6743 hospital admissions for ‘dog bites and strikes’ per year in England^5^ are a large underestimation of true incidence). Although in some senses this is reassuring that many dog bites do not cause significant physical injury, it is also known that even relatively minor bites can cause significant distress to the victim;^16^ thus they should not be considered unimportant to prevent. Perhaps multiple dog owners were more likely to complete the survey and inflate our dog bite incidences; however, the annual incidence of dog bites over the previous 12 months in our community was 18.7 per 1000 population which matches almost exactly population based studies in USA in the 1990s^10^ and 2000s.^32^ Our incidence of 1873 per 100 000 per year is nearly three times that of the 740 per 100 000 often cited but with no clear source.^11^ Regarding victim-level risk factors, our discovery that reporting being more emotionally stable is associated with reduced odds of having been bitten by a dog, is completely novel and unreported elsewhere. Males were also 1.6 times more likely to have been bitten than females and owners of multiple dogs were 3.3 times more likely to have experienced a bite than non-dog owners. Regarding our final aim, it was more common for participants to have been bitten by dogs completely unknown to them than familiar dogs.

This study has strengths and limitations. It only examined households in one geographical location and thus may not be generalisable to the wider population. It was part of a semirural small town, with a variety of housing types and socioeconomic factors.^27^ Within the survey population, 200 people (28.8%) reported currently owning a dog which is similar to estimations made by other studies conducted within UK.^1^ However, the respondents to the survey were proportionally more females (less likely to have been bitten) and older age groups (more likely to have been bitten). Furthermore, under 5 s, thought to be a high risk population, were not surveyed. In addition, the North-West of England experiences the highest rates of hospital admissions due to dog bites in the UK.^5^ Thus there are a number of reasons why our findings in terms of prevalence and incidence could have been both overestimated but also underestimated.

However, this study provides the first UK-based investigation of prevalence, incidence and risk factors for dog bites at a community population level and has significant advantages over hospital attendance data sources. One limitation of the data collected regarding the circumstances of the dog bite is that the respondent was asked to choose one bite event to describe if they had experienced multiple bites. However, a substantial number of bite victims within the study reported they had only been bitten once (57.6%), therefore it was possible to investigate the potential for bias by comparing relationships based on all dog bites with those based on individuals that had been bitten once and none was found. It is unknown whether the proportion of hospital admissions for dog bites, or even the actual rate of dog bites, has changed over the years, and as these data were collected retrospectively covering a number of years, it is unknown if it had an effect. While assessing bites retrospectively may lead to some details of the event being forgotten, it is unlikely that this would have affected the relatively simple questions regarding the bite required within this study. Recall problems are most likely when there is little time to retrieve information, the activity is common and timing is required to be recalled accurately.^33^ Dog bites are relatively uncommon over a lifetime, may be emotionally significant and we did not require specific timing of the event except for an indication of whether it had occurred in the past year.

Males were more likely to be bitten than females, which supports evidence from other studies at least in children.^34–36^ It may be that men receive more serious injuries and so are more likely to appear in hospital data; however, there was no evidence here that men were more likely to require medical treatment than women. It could be hypothesised that personality variation between genders has resulted in more males being subject to dog bites. Reporting being less emotionally stable was associated with an increased frequency of dog bites and so was being male. However, the multivariable analysis conducted adjust for the effects of these variables independently, and males were more likely to rate themselves as being more emotionally stable (data not shown). Negative indicators of emotional stability include descriptors such as insecurity, fear, instability and intrusiveness.^37^ Neuroticism (the other name for emotional stability) is well known to be linked to public health behaviours and outcomes;^38 39^ perhaps then this aspect of personality may be linked to performing behaviours that provoke dog bites. However, perhaps any association is not causal, in that people that have been bitten become more nervous or the association is being confounded by another factor such as socioeconomic status. Previous research has outlined mental disorders^40^ and attention deficit hyperactivity disorder in children^41^ as risk factors for dog bites. Another study found that less shy children took greater risks with a therapy dog.^42^ This adds strength to our finding that personality may be associated with dog bite incidence. There also is  evidence of an association between parent personality and child parenting style,^43^ which appears to echo in links between owner personality and dog personality^44^ and the nature of the dog–owner relationship.^45^ These studies suggest that nervous/anxious owners may have nervous/anxious dogs, which may be another explanation for increased bite risk. Much more research into the possible association with personality is now required, especially in order to understand if and how this knowledge could be used in dog bite prevention.

Many previous studies report that individuals are more commonly bitten by dogs that are familiar to them;^15 23 46^ however, this study has demonstrated that 54.7% of bites were by dogs the victim had never before seen. One hypothesis that could explain this difference is a possible bias towards people seeking medical treatment if bitten by a dog known to them,^35 46^ resulting in these types of dog bites appearing in hospital data. However, there was no evidence in our study that relationship to the dog varied with whether or not they received medical treatment. It may be that the association is present but not detectable given the study size.

Many of the historical bites reported occurred to individuals when they were young; however, only 3 of the 48 current children within the study (6.3%) had been bitten. This could indicate that childhood bites are becoming less common than they once were. Reduction of children playing outdoors in the modern era may be reducing the incidence of dog bites in children, in particular by strange dogs.^47^ The relatively high proportion of bites from unfamiliar dogs reported by participants in this study could be related to reporting of bites that occurred many years ago in childhood when free-roaming dogs were more commonplace. The relationships between risk of dog bite, age at bite and relationship to the dog require further investigation in a larger sample.

Multiple dog owners have previously been found to be at increased risk of dog bites,^32^ as here. It could be hypothesised that owners of multiple dogs may be a greater risk due to breaking up dog fights^8^ or simply having greater exposure to dogs and is a recommended area for further research, for example: are these bites from their own dog? are dog fights more likely? do these persons have increased exposure to other dogs in general? are multiple dog owners more likely to work in dog-related professions?

Our proportion of bites requiring hospital admission (0.6%) was very low and proportion requiring any medical treatment (33%) was higher rather than lower than the 19% reported in USA.^32^ Potential bias introduced into our estimate from those bitten multiple times is likely to be towards choosing to report to us the more serious injuries than less serious, meaning our proportion of bites described requiring hospital treatment may be underestimated. In fact, the proportions requiring medical treatment were similar even if someone had only been bitten once so we can be confident in our estimate in this respect. However, it must be noted that these statistical estimates is based on one community survey in one geographical area and thus it is questionable whether it can be generalised to the entire of England and certainly requires repeating to validate our proportions requiring medical treatment found.

Thus, future research should be conducted to corroborate our findings in other UK populations in order to assess the public health impact of dog bites and the significance of severity of injury. In particular, the causes and contexts of higher risk in owners of multiple dogs require further investigation and establishment of the repeatability and nature of the relationship between victim level of emotional stability/neuroticism and dog bite risk. Future studies should also investigate in more detail the contexts and situations regarding being bitten by familiar and unfamiliar dogs.^48^ Research is also required to estimate dog bite risk in under 5 s, which our study did not address. Our study also did not address any factors associated with the type of dog at risk of biting, such as sex, age and breed, which require more investigation as findings have been inconclusive.^49^

In conclusion, this study demonstrates that the most severe dog bites, of highest public health significance, are thankfully a small proportion of overall bites that occur. However, many more bites occur than present for treatment at a hospital and community level dog bite statistics such as those presented here are important to collect to validate our findings here. Unlike medical records-based studies, community survey data are likely to identify additional bites not requiring medical treatment and which are of unknown concern to the victim and whose public health significance is unclear. Further, this study has found that dog bite analysis based on hospital data does not always reflect the relationships seen when assessing population-based surveys. To better understand dog bites, future research should attempt to explore the circumstances of dog bites, the nature of the injury and victims’ perceptions and impacts on them. It is essential that previously assumed risk factors are reassessed as this study has revealed that prior beliefs, such as bites typically being from familiar dogs, are contested. Dog bite prevention initiatives may need to rebalance their target to include strange dogs with equal importance, as opposed to an emphasis on family pets in the home. Our study confirms that male victims are at increased risk and should be targeted for prevention. Dog bite prevention schemes may also need to target particular behaviours around dogs by different victim personality types. If UK or elsewhere hope to reduce dog bite incidence, it is essential that risk factors are accurately assessed in order to impose effective and well-informed dog policies in the future.

**What is already known on this subject**

- Dog bites are a public health problem of unknown quantity.
- It is thought that people are more often bitten by familiar dogs.
- Males are at higher risk of dog bites.

**What this study adds**

- An estimate of approximately 19 dog bites per 1000 population per year and only a very small proportion require hospital admission.
- Lower emotional stability may be a risk factor for having been bitten by a dog.
- Bites from unfamiliar dogs (55%) are common.
