# Supplementary material for: How many people have been bitten by dogs? A cross-sectional survey of prevalence, incidence and factors associated with dog bites in a UK community
Source: J Epidemiol Community Health. 2018 Feb 1;72(4):331–6. doi: 10.1136/jech-2017-209330 (PMC5868524; doi:10.1136/jech-2017-209330)
Supplement: Supplementary file [file jech-2017-209330supp001.pdf]

How many people have been bitten by dogs?: A cross-sectional survey of prevalence, incidence, and factors associated with dog bites in a UK community

Carri Westgarth, Megan Brooke, Robert Christley

Supplementary file – Univariable analysis of factors associated with having ever been bitten by a dog

**Variable**		**N**	**Bitten by dog (n, %)**	**Not bitten by a dog (n, %)**	**OR**	**95%CI**	**P**
Gender	Female	366	77 (21.0%)	289 (79.0%)	1.00		
	Male	314	95 (30.3%)	219 (69.7%)	**1.63**	**1.15 – 2.31**	**0.006**
	Missing		0	0			
Current dog ownership	Non dog owner	482	111(23.0%)	371 (77.0%)	1.00		
	Dog owner	198	61 (30.8%)	137 (69.2%)	**1.45**	**1.03 – 2.13**	**0.03**
	Missing		0	0			
Number of dogs	Owns no dogs	482	111(23.0%)	371(77.0%)	1.00		**0.05**
	Owns one dog	174	51(29.3%)	123(70.7%)	1.39	0.94 – 2.05	
	Owns multiple dogs	24	10 (41.7%)	14(58.3%)	**2.39**	**1.03 – 5.52**	
	Missing		0	0			
Dog walking	Not main dog walker	59	18 (30.5%)	41 (69.5%)	1.00		
	Main dog walker	130	42 (32.3%)	88 (67.7%)	1.04	0.56 – 2.11	0.81
	Missing*		0	0			
Age	Years	668	Median - 59	Median 57.5			0.32
	Missing		1	11			
Highest Education	Other school certificate or none	97	23 (23.7)	74 (76.3)	1.00		0.28
	GCSE or O'level equivalent	146	44 (30.1)	102 (69.9)	1.39	0.77-2.50	
	A level equivalent	67	12 (17.9)	55 (82.1)	0.70	0.32-1.53	
	Degree/diploma or higher professional qualification	294	77 (26.2)	217 (73.8)	1.14	0.67-1.95	
	Missing*		6	58			
General health	Excellent	92	18 (19.6%)	74 (80.4%)	1.00		0.10

	Very good	218	60 (27.5%)	158 (72.5%)	1.56	0.86 – 2.83	
	Good	240	51(21.3%)	189 (78.8%)	1.11	0.61 – 2.023	
	Poor	31	10 (32.3%)	21 (67.7%)	1.958	0.786 – 4.88	
	Fair	92	30 (32.6%)	62 (67.4%)	**1.989**	**1.01 – 3.91**	
	Missing		3	4			
Reason for getting a dog	Companionship - Yes	116	36 (31.0%)	80 (69.0%)	1.00		
	No	73	24 (32.9%)	49 (67.1%)	1.01	0.58 – 2.04	0.79
	Family wanted- Yes	72	17 (23.6%)	55 (76.4%)	1.00		
	No	117	43 (36.8%)	74(63.2%)	1.88	0.97 – 3.64	0.06
	Exercise – Yes	64	21 (32.8%)	43 (67.2%)	1.08	0.566 – 2.05	
	No	125	39 (31.2%)	86 (68.8%)	1.00		0.82
	Always had dog – Yes	58	20 (34.5%)	38 (65.5%)	1.20	0.62 – 2.31	0.56
	No	131	40 (30.5%)	91 (69.5%)	1.00		
	Interest - Yes	33	13 (39.4%)	20 (60.6%)	1.51	0.69 – 3.28	0.30
	No	156	47 (30.1%)	109 (69.9%)	1.00		
	Other - Yes	12	6 (50%)	6 (50%)	2.28	0.70 – 7.38	0.16
	No	177	54 (30.5%)	123 (69.5%)	1.00		
	For protection – Yes	11	4 (36.4%)	7 (63.4%)	1.25	0.35 – 4.43	0.74
	No	178	56 (31.5%)	122 (68.5%)	1.000		
	Gift- Yes	6	2 (33.3%)	4 (66.7%			
	No	183	58 (31.7%)	125 (68.3%)			
	Working Dog- Yes	5	2 (40%)	3 (60%)			
	No	184	58 (31.5%)	126 (68.5%)			
	Show Dog- Yes	1	1 (100%)	0 (0%)			
	No	188	60 (31.9%)	128 (68.1%)			
	Missing*		0	0			
Personality Trait	Extroverted	581	Mean – 4.21	Mean - 4.28	0.97	0.86 – 1.09	0.61
	Missing*		13	38			
	Conscientiousness	584	Mean – 5.71	Mean – 5.76	0.96	0.80 – 1.14	0.40
	Missing*		14	34			
	Open to new experiences	582	Mean - 4.95	Mean – 4.88	1.06	0.90 – 1.23	0.586

	Missing*		14	36			
	Agreeableness	582	Mean – 5.34	Mean - 5.40	0.95	0.81 – 1.12	0.434
	Missing*		14	36			
	Emotional stability$	585	Mean – 5.01	Mean – 5.11	**0.82**	**0.72 - 0.94**	**0.005**
	Missing*		13	34			

*not all of the 680 people who responded on whether they had been bitten were asked this question

$ Emotional stability also known as neuroticism
